# AFM Monitoring the Influence of Selected Cryoprotectants on Regeneration of Cryopreserved Cells Mechanical Properties

**DOI:** 10.3389/fphys.2018.00804

**Published:** 2018-06-29

**Authors:** Martin Golan, Sarka Jelinkova, Irena Kratochvílová, Petr Skládal, Martin Pešl, Vladimír Rotrekl, Jan Pribyl

**Affiliations:** ^1^Department of Analysis of Functional Materials, Institute of Physics, Academy of Sciences Czech Republic, Prague, Czechia; ^2^Department of Biology, Faculty of Medicine, Masaryk University, Brno, Czechia; ^3^International Clinical Research Center, St. Anne's University Hospital, Brno, Czechia; ^4^Central European Institute of Technology, Masaryk University, Brno, Czechia; ^5^First Department of Internal Medicine/Cardioangiology, Masaryk University, Brno, Czechia

**Keywords:** cryopreservation, cell stiffness, AFM, fluorescence microscopy, DMSO, PEG

## Abstract

Cryopreservation of cells (mouse embryonic fibroblasts) is a fundamental task for wide range of applications. In practice, cells are protected against damage during freezing by applications of specific cryoprotectants and freezing/melting protocols. In this study by using AFM and fluorescence microscopy we showed how selected cryoprotectants (dimethyl sulfoxide and polyethylene glycol) affected the cryopreserved cells mechanical properties (stiffness) and how these parameters are correlated with cytoskeleton damage and reconstruction. We showed how cryopreserved (frozen and thawed) cells' stiffness change according to type of applied cryoprotectant and its functionality in extracellular or intracellular space. We showed that AFM can be used as technique for investigation of cryopreserved cells surfaces state and development *ex vivo*. Our results offer a new perspective on the monitoring and characterization of frozen cells recovery by measuring changes in elastic properties by nanoindentation technique. This may lead to a new and detailed way of investigating the post-thaw development of cryopreserved cells which allows to distinguish between different cell parts.

## Introduction

The aim of cryopreservation is to reanimate frozen cells to physiological life with negligible loss of viability and functionality. Effective cryopreservation is an important problem in medicine (Woods et al., 2016), pharmaceutical, food industries, and agriculture. A major drawback of cryopreservation is that ice crystallization appearing during the freezing process can significantly damage the cells which then lose viability after melting (Chen et al., 2013; Rajan et al., 2016; Ding et al., 2017). The fact that big part of cells from a multitude of prokaryotic and eukaryotic organisms can be recovered from temperatures down to almost −200°C below the freezing point is thus achieved due to a trick—the presence of cryoprotectants. A multitude of factors affect the effectiveness of cryopreservation in microorganisms, for example, species, strain, cell size and form, growth phase and rate, incubation temperature, growth medium composition, pH, osmolarity, cell water content, lipid content and composition of the cells, density at freezing, composition of the freezing medium, cooling rate, storage temperature and duration of storage, warming rate, and recovery medium (Hubalek, 2003; Kratochvilova et al., 2017).

Based on the extent to which the cryoprotectant is able to enter into the cell, three categories of additives can be distinguished: (1) cryoprotectants penetrating both cell and nuclear membrane [dimethylsulfoxide (DMSO), glycerol]; (2) cryoprotectants penetrating cell membrane but not nuclear membrane (mono-and disaccharides, amino acids); (3) cryoprotectants not penetrating even cell membrane (proteins, polysaccharides, PEG-1500 where 1500 denotes molecular mass).

The type of cryoprotectant is usually defined according to its cell penetration depth and speed. Quickly penetrating cryoprotectants [e.g., ethylene glycol, propylene glycol, dimethylsulfoxide (DMSO)] usually permeate the whole cell volume within 30 min. The permeation rate of glycerol is comparatively slower. Non-permeating cryoprotectants [polyvinyl pyrrolidone, PEG-1500, or polyvinyl alcohol (PVA)] cannot enter the cell due to their high molecular mass (Hubalek, 2003).

The studies of changes in surface characteristics of cell membranes caused by freeze-thawing can be important for assessment of structural and functional integrity of cells and understanding the mechanisms responsible for their damage under extreme conditions (Mandumpal et al., 2011). Deleterious changes in cytoskeletal component such as disruption of the actin filaments were observed in thawed cells. This damage was caused mainly by the freezing process itself (Ragoonanan et al., 2010; Xu et al., 2012). This disruption of actin filaments was identified to be caused by defective F-actin polymerization after freezing process (Chinnadurai et al., 2014).

It has been demonstrated that cell mechanical stiffness is mainly determined by the cytoskeleton, especially the networks of actin and intermediate filaments and other proteins associated with them. And thus disruption and other remodeling processes could contribute to cell stiffness changes. Among the many devices for microindentation (Levental et al., 2010), the Atomic Force Microscope (AFM) is commercially available and has been widely applied to characterize mechanical properties of living cells and tissues (Wang et al., 2009). AFM (Alessandrini and Facci, 2005; Masek et al., 2011a,b; Fekete et al., 2012; Cartagena-Rivera et al., 2015; Pesl et al., 2016) allows to scan living cell topography under ambient conditions (liquid medium), and also offers a force spectroscopy mode. In this mode, cell is indented at many sites and its complete elastic response is recorded which enables to reconstruct its stiffness map (Ogden, 1972).

Using AFM we studied changes of mechanical properties of cells resulting from their exposure to cryogenic temperatures after application of cryoprotectants either penetrating both cell membrane and nuclear membrane (DMSO) or penetrating neither of these membranes (PEG 1500) (Dokukin et al., 2013; Guz et al., 2014; Gavara, 2016; Dokukin and Sokolov, 2017). These factors have an impact on cellular structure, which in turn influences cell elasticity (Ofek et al., 2009; Masek et al., 2011a).

By combination of AFM and fluorescence microscopy we showed how selected cryoprotectants (DMSO, PEG) affected mechanical properties and cytoskeleton remodeling of cryopreserved cells (mouse embryonic fibroblasts). Significant differences of the investigated properties of the thawed cells were found as a response to the cryoprotectant used in the freezing process.

## Materials and methods

### Cell culture, freezing, and sample preparation

Mouse embryonic fibroblasts (MEFs) (CF-1 mouse strain; Eiselleova et al., 2008) were propagated on 100 mm Petri dish in MEF medium [MEF medium consists of Knockout Dulbecco's modified Eagle's medium (KO-DMEM; Gibco), 10% heat-inactivated fetal bovine serum (FBS; Invitrogen), 1% L-glutamine (Gibco), 1% non-essential amino acids (PAA), 1% penicillin-streptomycin (PAA), and 0.1 mM β-mercaptoethanol (Sigma)] until passage 2 (P2). A confluently grown cell culture was incubated with SIR F-Actin (Spirochrome, distributed by tebu-bio, Offenbach, Germany) in concentration of 1 μM, and verapamil in concentration 1 μM for 90 min. The cells were washed with PBS and trypsinized (Trypsin EDTA, Invitrogen, Ca USA) for 2 min and collected into centrifuge tube. The cell suspension was spinned by 200 g/4 min and resuspended to concentration 1,000,000 cells/ml. 200,000 cells (in 0.2 ml of MEF medium) were placed into 2 ml cryo tube (TPP, Trasadingen, Switzerland). Freezing was done by addition of 0.2 ml of ice-cold freezing medium drop by drop into the tube.

Variable freezing media were used in final concentrations as follows: DMSO freezing medium consisting of 80% KO-DMEM, 10% FBS, and 10% DMSO (Hybri-Max™, Sigma); PEG1500 freezing medium consisting of 82.5% KO-DMEM, 10% FBS, and 7.5% polyethylene glycol (Mw 1500; PEG1500; kind gift from Dr. Karel Pomeisl, Institute of Physics, Academy of Sciences Czech Republic). The tubes were placed in Nalgene dish for 24 h in −80°C, then transferred to liquid nitrogen (at least for 24 h) until measurement.

Cryo tube with MEFs P2 was taken out from liquid nitrogen (Dewar flask) and cell suspension was quickly thawed in stream of hot water until the point, where a few remaining crystals were visible in the tube. The whole volume was immediately transferred into the 15 ml centrifuge tube and 12 ml of cold (4°C) MEF medium was added dropwise from Pasteur pipette. The tube was centrifuged (200 g, 4°C) for 4 min, the excess of MEF medium above the cell pellet was removed by pipette, cells were resuspended in 1 ml of MEF medium (37°C) and 0.5 ml distributed onto 35 mm low ibidi dish (ibidi GmbH, Martinsried, DE) for AFM measurements and 0.5 ml into 35 mm Glass Bottom Culture Dish (MaETek corporation, Ashland, MA, USA) for Life Imaging procedures. The dishes were placed into standard CO_2_ incubator for 20 min for the cells to attach. For AFM measurements, all unattached cells from ibidi dish were removed by PBS wash and fresh MEF medium was added into the dish with 100 nm mol/l of SIR F-Actin, installed in the AFM dish holder pre-heated during the calibration process and after the AFM instrument adjustment, the force mapping measurement was immediately started.

### Young's modulus mapping by atomic force microscope

Standard bio AFM microscope JPK NanoWizard 3 (JPK, Berlin, Germany) was used to perform force mapping procedure. The scanning-by-probe head (maximal visualization range 100–100–15 μm in X-Y-Z axis) of the AFM system was placed on inverted optical microscope Olympus IX-81 (Olympus, Tokyo, Japan), 10x objective was used to find proper area covered with cells and to place cantilever in the proper position for the force mapping procedure. Plastic Petri dish with either the distilled water for instrument calibration or with the fibroblast culture was placed inside the Petri dish heater (JPK) pre-heated to 37°C.

We compared set points, indentation depths and the Young's modulus (YM) of cultivated cells for measurements performed by the spherical and pyramidal AFM tips, respectively. For spherical tip and small set points (e.g., lower than 0.5 nN) the contact parts of force curves were not sufficient for reliable fits. On the other hand, force curves obtained with pyramidal tip and higher set point values (1.5 nN and above) were often not smooth. The notches observed on the curves likely corresponded to structural damage of the measured cells.

When using a spherical probe at high (1 nN) set point, cells were indented down to similar depth as when using the pyramidal probe at low set point (lower than 0.5 nN) with close values (deviation less than 8%) of obtained Young's modulus. Based on these considerations, we chose to use the spherical probe with set point 1 nN. Thus, the elastic properties of the cells could be probed down to significant indentation depths (~0.5–1 μm) while avoiding structural damage to the measured cells. Furthermore, the obtained YM values were less susceptible to variations caused by small-scale membrane and submembrane features thanks to the large contact surface of the colloidal probe (as opposed to the pyramidal tip). Non-coated silicon nitride cantilevers with colloidal sphere probe made of hydrophilic silicon dioxide (diameter, 6.62 μm) sCUBE CP-sq-SCONT-SiO-C (sQUBE, Bickenbach, Germany) were used for experiments with spherical indenter. Non-coated silicon nitride AFM cantilever Hydra 2R-100N (AppNano, Mountain View, CA, USA) equipped with silicon pyramidal probe (side angle 18°) were used in the optimization study, i.e., to compare pyramidal and spherical probe indentation. The probe was calibrated prior to every experiment as described below.

The calibration procedure was done in double distilled water, when the whole setup was pre-heated (Petri dish heater) to 37°C for 30 min. Then the laser reflection sum was maximized, followed by centering of the laser detector. The AFM probe was introduced in contact with the surface during a standard process of landing. The sensitivity of the AFM setup was determined as a slope of the force-distance curve measured by lifting the cantilever with Z-height of 450 nm, time per curve was 3 s. The sensitivity was found in the range 15.07-15.37 nm/V, cantilever stiffness was calibrated by measurement of its thermal noise and lay between 17.34 and 19.19 mN/m for different days of experiments.

The bio AFM setting was identical for all the force mapping procedure. Set Point value was 1.0 nN (relative to baseline value), time per curve 0.45 s, Z-length 15.0 μm, speed of curve recording 33.3 μm/s, the force-distance curves were recorded with data sample rate of 2 kHz. The force mapping procedure was performed as step-by-step recording of force-distance curves in the network of 64 × 64 points on 75 × 75 to 100 × 100 μm^2^ covering area of either single or more fibroblast cells.

Reproducibility of the nanomechanical measurement was performed by 5-times repeated force mapping process on identical place (scanning over the identical cell). Neither medium exchange nor AFM instrument adjustment was involved during the repeated mapping procedure.

Place-to-place reproducibility was studied, when the identical force mapping experiments were subsequently performed on two different places found randomly on the Petri dish surface covered with fibroblasts.

Before the measured AFM data were further processed and interpreted, all parts of each sample corresponding to the plastic dish were algorithmically removed using Wolfram Mathematica (Wolfram, 2017).

The fluorescence stack images were recorded periodically between the capturing of the force maps, when the AFM detection laser was switched off and the AFM indenter was not in the contact with the surface, however the AFM measuring head was kept in the position to be able to start another indentation process immediately. The fluorescence images of the SiR-actin stained cells were recorded using the Olympus IX-81S1F-3 inverted microscope equipped with U-MF2 ET-CY5 filtration cube (Olympus) and mercury lamp Olympus U-RFL-T. The stack images were recorded using the Andor Zyla 5.5 sCMOS camera (Andor, Belfast, UK). Both, inverted microscope and sCMOS camera, were driven by Olympus CellSens Dimension software. This software was used to record and post-process the stack images—i.e., a series of images taken in different focal length, typically 15–25 images were recorded with step of 0.23 μm). The stack images were afterwards combined to produce final image showing the structure of cytoskeleton.

### Combined monitoring of the post-thawing properties of the cryopreserved cells

Surface stiffness of the fibroblasts cultured on a Petri dish 30 min after thawing process was monitored till 4.5 h after thawing by nanomechanical mapping of the fibroblast cell. The scanned area was not changed during the whole monitoring. The AFM measurements were combined with fluorescence imaging.

In detail, Petri dish containing freshly thawed suspension of cells was pre-incubated in a standard CO_2_ incubator. When first cells started to adhere (30 min after thawing), culturing medium in the dish was completely exchanged and fluorescence stack imaging (see previous chapter for details) periodically followed by force mapping process was started immediately.

Culturing medium inside the dish was changed for the new one after each force-mapping procedure, when the tip was not in contact with the sample surface. Following parameters were used for the FM procedure: Setpoint value was either 0.75 nN for pyramidal tip or 1.0 nN when the spherical indenter was used. Time per curve was 0.5 s, Z-length 15.0 μm, speed of curve recording 30.0 μm/s, the force distance curves were recorded with data sample rate of 2 kHz. The force mapping procedure was performed as step by step recording of force distance curves in the network of 64 × 64 points on 75 × 75 to 100 × 100 μm^2^ covering area of either single or more fibroblast cells.

Force mapping process provides a network of force-distance curves (FDC, dependency of tip-sample interaction force on tip height above the surface), so called force maps (FM) (Dimitriadis et al., 2002; Touhami et al., 2003). The absolute value of Young's modulus can be determined by fitting the FDC by equation 1 (spherical probe, Hertz-Sneddon model; Sneddon, 1965; Gavara and Chadwick, 2012).

(1) $$\begin{array}{r} P=\frac{E\;}{\;\left(1-\upsilon^{2}\right)}\left(\frac{R^{2}+a^{2}}{2}Log\left(\frac{R+a}{R-a}\right)-aR\right),\\ \delta =\frac{a}{2}Log\left(\frac{R+a}{R-a}\right) \end{array}$$

Where *P* is load, *E*—Young's modulus, ν—Poisson ration (0.5 for incompressible materials), δ—depth of indentation; a—contact radius, *R*—radius of the spherical probe.

The fitting of FDC by Equation (1) was performed in the AtomicJ software (Hermanowicz et al., 2014), with the contact point position estimated by an incorporated Robust Exhaustive algorithm and best fit found by the Robust HLTA algorithm. Poisson ratio was set to 0.5. In all cases, the fitting was performed on the approach curve. Resulting Young's modulus maps were exported in order to be post-processed in Wolfram Mathematica (Wolfram, 2017).

After the fitting, some points were removed from the YM maps based on the values of various parameters of the corresponding fit. First, a threshold of 10 kPa was introduced for the YM value in order to exclude all curves measured over the dish surface or over very thin cell regions.

Furthermore, all fits yielding indentation greater than 2 μm were filtered out as such a large indentation always meant a faulty contact point estimation. Here, indentation was defined as the indentation depth difference between the shallowest (i.e., contact) point and the deepest point of the fitted part of the FDC.

Also, if the indentation force at the deepest point of the fitted region was less than 80% of the total set point, the corresponding curve was removed. Thus, FDCs whose fitted region was too small were not taken into consideration.

Among other benefits, these constraints helped us to exclude force curves which couldn't be well-described by the Hertz-Sneddon model with single *E* value (i.e., they typically contained a significantly stiffened region at larger depths). Such curves were typically located in the border regions of the measured cells. In the remaining curves, it was therefore not necessary to employ modified indentation models incorporating e.g., the bottom effect cone correction (Gavara and Chadwick, 2012).

Finally, the adequacy of the Hertz-Sneddon model was checked. The curves kept for final statistical analysis had root-mean-square deviation of the model from the actual data points smaller than 5% of the maximum set point, and at each point the maximum deviation of the model values from the measured data was always smaller than 7% of the maximum set point. After applying all filters, 90% of curves measured on cells and corresponding YM values were left for statistical analysis.

### Live imaging

The thawed cells were left to attach to the culture dish for 30 min, after the complete exchange of medium, the dish was left for additional 10 min in the incubator then transferred onto inverted confocal Zeiss LSM700 microscope with 37°C and 5% CO_2_. Time Series video was taken with 3 min interval for 120 cycles (6 h) on 40x Oil immersion objective, with laser intensity 1.8%, pinhole 228.6 (6.2 μm) and samples were excited with 639 nm laser and fluorescence detected in far red spectrum (for actin labeling) and in phase contrast (for cell morphology) (acquisition speed 25–30 s per image). Videos were managed and exported using ZEN Black or ZEN Blue system.

### Viability of cells

Flow cytometry was used to quantify survival and apoptosis in cells that were frozen with or without cryoprotectants. The Muse® Cell Analyser (Merck Millipore) and Muse® Annexin V and Dead Cell Assay Kit (MCH100105, Millipore), which can discriminate between live, early apoptotic, late apoptotic/necrotic and dead cells, were used according to Hofer et al. (2016).

The viability of the fibroblast cells was tested by standard TrypanBlue test. Time points of the test were selected to be identical with the force mapping procedure, i.e., viability was checked every 30 min, from 0.5 h till 4.5 h after thawing. The wells of standard microtitration plate were washed to exclude floating cells, trypsinized and collected into tubes. Cell suspension was then incubated in 0.5% TrypanBlue solution (1:1) for 2 min and viable cell ratio was counted on hematocytometer. For the testing of cell viability after freezing/thawing, 10 experiments were performed for each cryoprotectant.

### Statistical evaluation of data

For each cryoprotectant, 3 experiments were performed. Total number of mapped DMSO treated frozen/thawed cells was 9 because in some maps, multiple cells were present. Mapping of cells frozen/thawed in PEG-1500 was done on 8 cells. The normality of the distribution of *E* values obtained from different cells at a certain time point was evaluated by Shapiro Wilk method thus proving the data normality at 0.05 level. Standard error of the mean values for each time point was less than 7%.

After measuring the force curves across the whole area, each force curve was fitted with the Hertz-Sneddon model, which yielded the YM value. Then, we removed the YM values that resulted from a faulty (aforementioned) fit (or rather a fit of faulty curves which occasionally occurred in the set). In the remaining set of curves, we analyzed the distribution of YM values in different surface parts (upper and lower half) and also calculated mean and median of the whole cell YM.

## Results and discussion

Using flow cytometry, we first checked how the application of cryoprotectants (DMSO, PEG) affected cell viability. Both DMSO- and PEG-treated non-frozen cells had viability over 90% (Supplementary Table 1). Next, we measured cell viability of cells after freezing/thawing. Without cryoprotectants, almost all frozen cells died after being thawed; only <5% survived thawing. The highest cryoprotective effect was provided by DMSO (>80% thawing survival). The improvement of cell viability by PEG was also relatively large: close to 50% of cells survived thawing. Thanks to its small size and physical-chemical properties, DMSO is able to penetrate both into the cell cytoplasm and the nucleus where it protects cells against freezing damage very effectively (Dong et al., 2010). On the contrary, PEG 1500 due to its high molecular mass is not able to penetrate even cell membrane and protects the cell only from the extracellular space, which limits its cryoprotective efficiency.

The viability of the cells tested by AFM was measured at the time points from 30 min to 4.5 h after thawing (Supplementary Table 2) in order to correspond to force mapping procedures. During this whole time, the survival rate of DMSO-cryopreserved cells remained twice as high as the population of PEG-cryopreserved cells. For either treatment, the cell viability didn't significantly decrease during the AFM monitoring.

**Figure 1:**
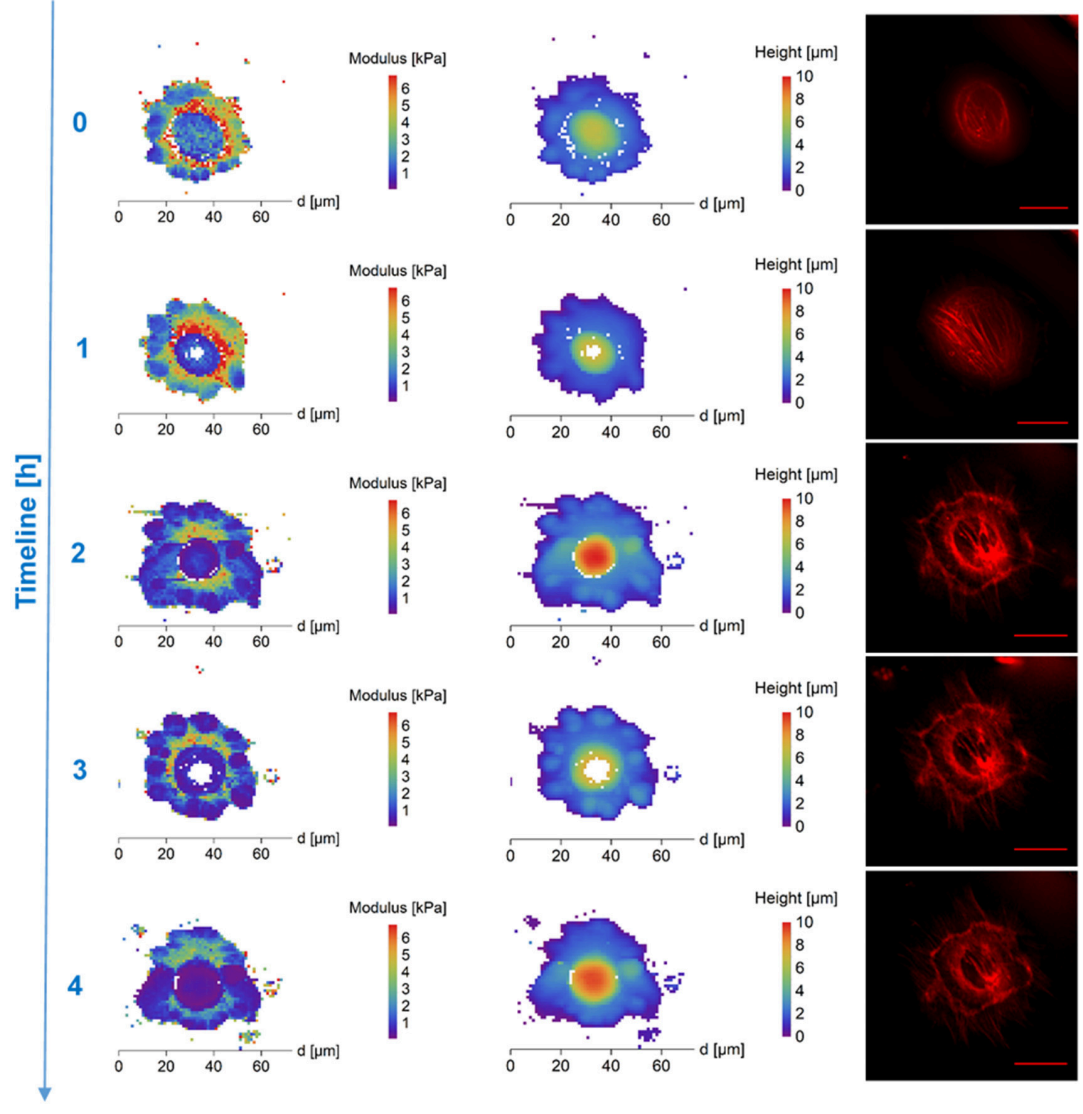
AFM maps of Young's Modulus **(Left)**, height (column of image in the **Middle**) and fluorescence images of cytoskeleton **(Right)** of DMSO treated frozen/thawed cells during monitoring of the post-thawing process. Each step shows a change of the cell biomechanical and structural properties during 60 min of the cell life. DMSO affected most significantly the cell's stiffness in the core region. The DMSO-treated cells had a softer core after thawing—compared to extra nuclear region/surrounding cytoplasm and clearly recognizable cytoskeleton in the map of thawed cells surface stiffness. Scale bar in the fluorescence images is equal to 30 μm.

In order to determine the cryopreservation properties of the selected cryoprotectants on the cytoskeleton remodeling upon thawing we measured the surface stiffness of cells treated with DMSO or PEG. By repetitive measurements we further observed the dynamics of the development of the cell surface stiffness in detail (Figure 1). In order to see the differential effect of cryoprotectants on different parts of the cryopreserved cells we correlated the surface stiffness maps and cell surface height (Figure 1). We distinguished two major regions, the core/nuclear region forming 50–100% of the total cell height and cell edges located outside the nuclear region which formed 0–50% of the total height of the cell. In order to project the changes in cell surface stiffness into the remodeling cytoskeleton we combined the AFM with standard fluorescence-based method for live cytoskeleton visualization which demonstrated that cell surface stiffness closely resembles the cell cytoskeleton regeneration occurring after freezing/thawing.

**Figure 2:**
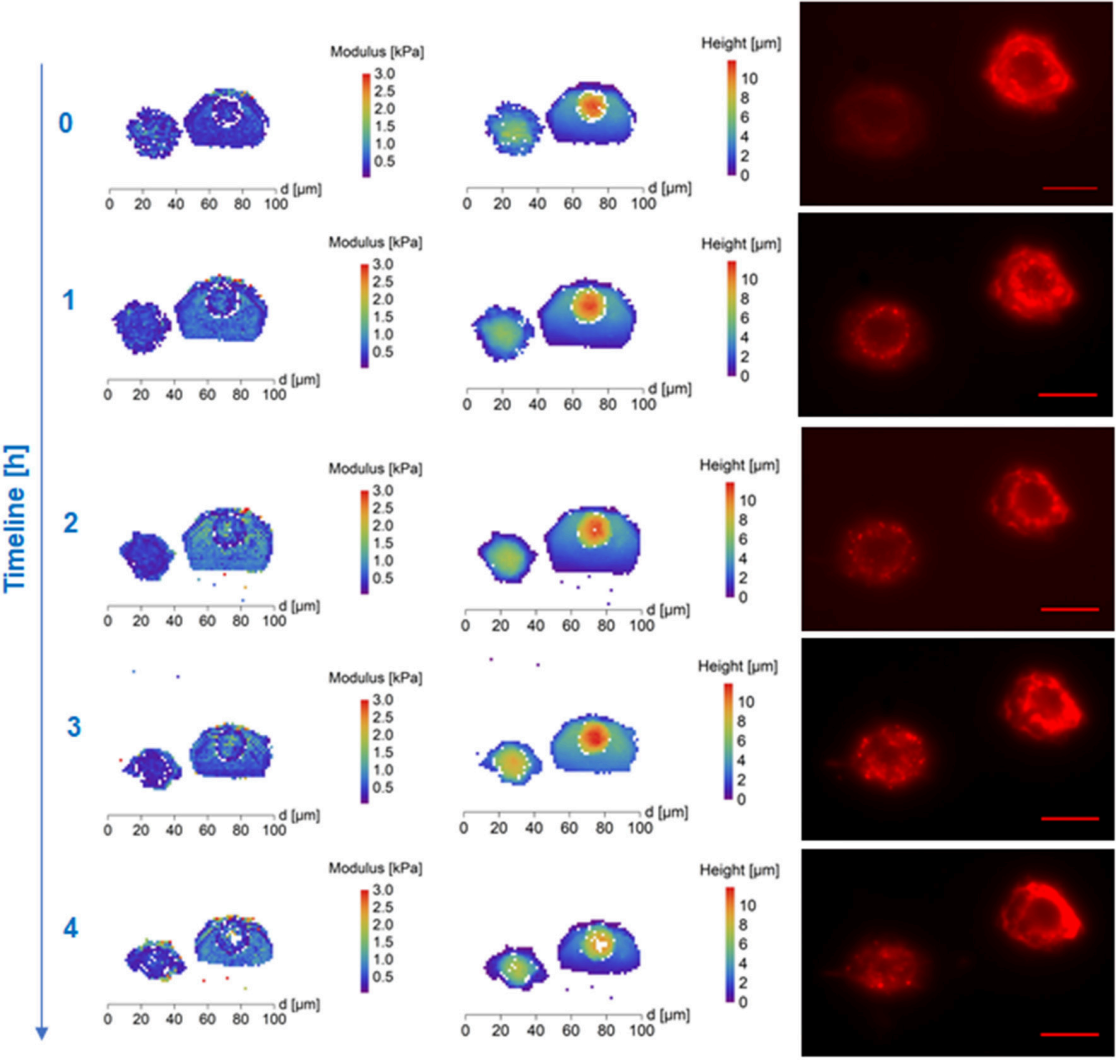
AFM maps of Young's Modulus **(Left)**, height (column of image in the **Middle**) and fluorescence images of cytoskeleton **(Right)** of polyethylene glycol (Mw 1,500, PEG1500) treated frozen/thawed cells during monitoring of the post-thawing process. Each step shows a change of the cell biomechanical and structural properties during 60 min of the cell life. Polyethylene glycol (Mw 1,500) treated cells both cytoskeleton and cell nuclei became softer and the height of the cell increased. Scale bar in the fluorescence images is equal to 30 μm.

DMSO affected most significantly the cell's stiffness in the core region. Approximately 30 min after thawing, the DMSO-treated cells had a softer nucleus (mean values)—compared to surrounding cytoplasm in the map of thawed cells surface stiffness with clearly recognizable cytoskeleton in fluorescent microscopy (Figure 1, Supplementary Figure 2). The stiff perinuclear area presents itself with a dense ring of structured actin that seems to correlate with observed higher stiffness. After forming of linear actin stress fibers (observed 70 min after thawing), the area with the highest density of these structures remains also the stiffest (Figure 1) as was also shown in fibrous cartilage samples where presence of fiber increases the local stiffness (e.g., Loparic et al., 2010; Kasas et al., 2013). That could be explained by the property of DMSO to bundle actin filaments into thick fibers (Lampugnani et al., 1987), which could be better protected against the very low temperatures. Gradual development of the round shaped structures at the border of the cells can be seen in case of DMSO cryoprotected fibroblasts (Figure 1, time points 0.5-4.5 h post-thaw). This may correspond to the development of lamellipodia, which are a part of the cell adhesion apparatus (Parsons et al., 2010). This is supported by the fluorescence images in Figure 1 which show an increasing amount of actin fibers. On the contrary, PEG-cryopreserved cells present more spheroidal shape after thawing and initial attaching, with rather homogeneous spatial distribution of stiffness (mean values). The stiffness of PEG-treated frozen/thawed cells nuclei are close to stiffness of the surrounding cytoplasm (mean values) (Figure 2, Supplementary Figure 3). That suggests decreased actin remodeling potential, since the actin cytoskeleton pattern is unstructured or broken, showed by homogeneous low stiffness of the whole cell area. The actin disruption could be caused by absent protection of cytoskeleton proteins inside the cells, since PEG is a non-penetrating chemical. Due to low temperatures, the actin structures are damaged (Ragoonanan et al., 2010; Pogoda et al., 2012; Xu et al., 2012) and expression of new actin proteins is affected too (Lin and Tsai, 2012) causing decline in regeneration of actin structures responsible for support to the cell membrane.

Time development of stiffness of cryopreserved cells reflects the cryoprotectant interaction with the cell and cryoprotective functionality of the material. The stiffness of PEG treated frozen and thawed cells was low (compared to DMO treated cells) and homogeneous over the whole cells. The stiffness of PEG treated cells change in time is relatively (compared to DMSO treated cells) mild (mean values changes) after thawing. The time required for proper attachment and forming of linear stress fibers significantly differ between DMSO and PEG medium. The time required for flattening of the cell, which was accompanied by change of dense ring structure around the nucleus remodeled into first linear fibers of actin was 27 ± 15 min in DMSO (10 cells) and 53 ± 24 min in PEG1500 (5 cells) freezing medium. This corresponds to the measured greater height of the PEG frozen cells and constant lower elasticity as the cytoskeleton is remodeling slowly into fibers. While DMSO frozen cells create a dense actin ring around the nucleus after plating, which increases stiffness in the perinuclear area in correspondence to actin fiber presence, the non-structured filaments in PEG frozen cells have no effect on stiffness elevation of the cell and thus the whole cell is softer. These data correspond to the possible DMSO protection function of actin fibers (Lampugnani et al., 1987) which start to remodel soon after thawing. Conversely, these data suggest a lack of protection by PEG, leading to actin fiber disruption and a slowdown of initiation of new actin protein expression caused by the freezing process (Lin and Tsai, 2012). Development of the cytoskeleton in a real time was monitored by confocal microcopy for period of 5 h and can be seen as videos in the Supplementary Material section (video captions are shown as Supplementary Figure 1).

**Figure 3:**
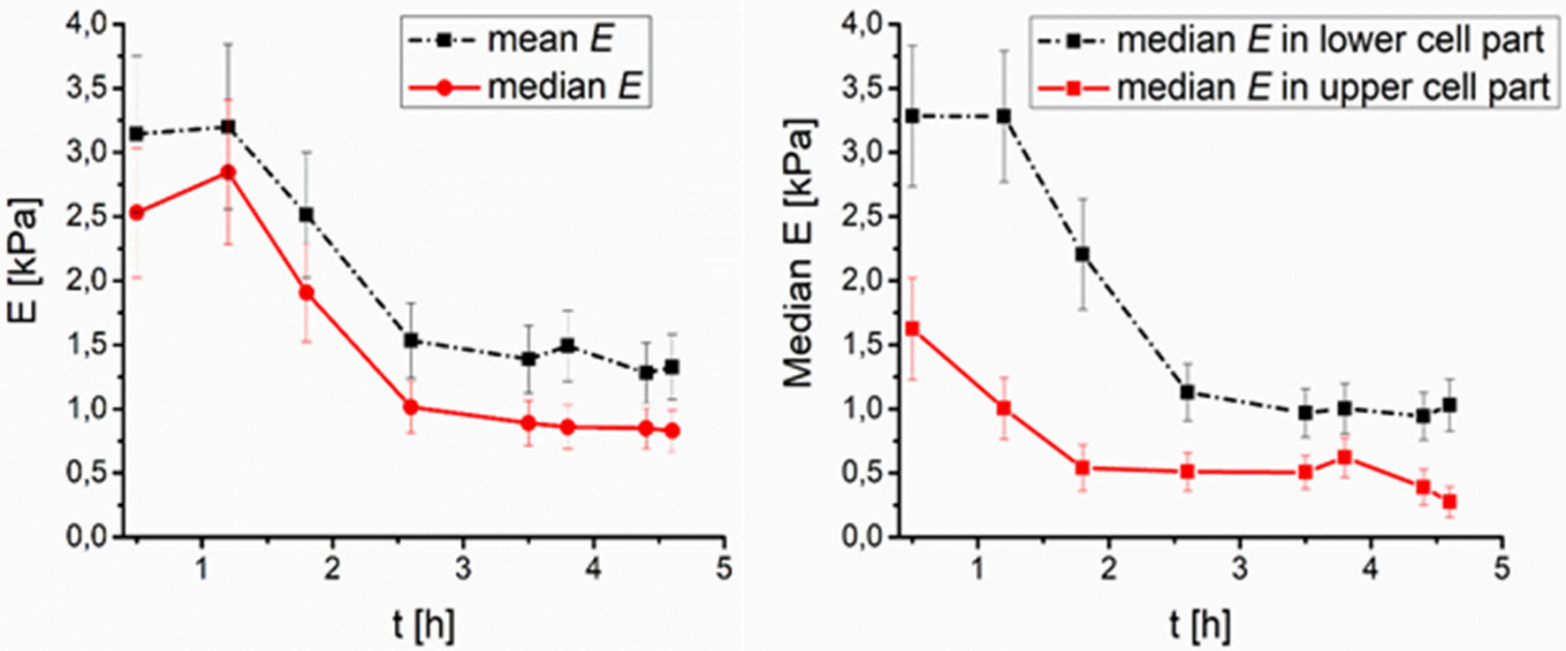
Time development of DMSO treated fibroblasts surface elasticity median after thawing (number of cells *N* = 9). **(Left)** Median and mean value of elasticity over time, **(Right)** median value of elasticity in lower (0–50% of the full cell height), and higher (50–100% of the full cell height) part of cell over time.

**Figure 4:**
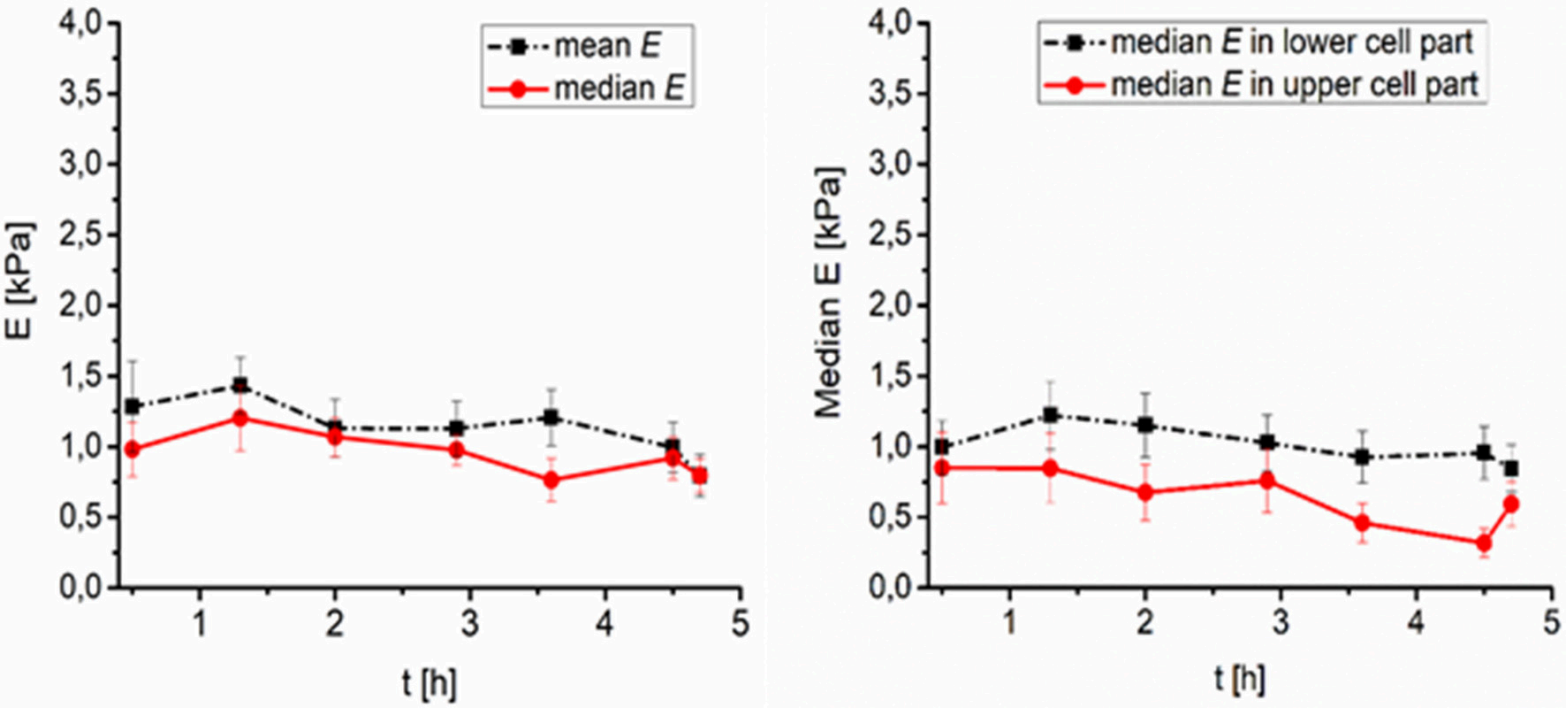
Time development of PEG treated fibroblasts surface elasticity (Young's modulus E) after thawing (number of cells *N* = 8). **(Left)** Median and mean value of elasticity over time, **(Right)** median value of elasticity in lower (0–50% of the full cell height), and higher (50–100% of the full cell height) part of cell over time.

As far as cell stiffness time development after thawing is concerned, the stiffness of DMSO-cryopreserved cells significantly decreases during first 2 h (up to 60%), while the stiffness change of the PEG1500-cryopreserved cell remains low (maximum decrease of 20%) even till 4.5 h after thawing (Figures 3, 4). For DMSO or PEG treated frozen and thawed cells the values of *E* from the core part of the cell had the smallest spatial deviation from median *E*–less than 10%. Such deviation corresponds to relatively sharp distributions of *E* values (within core regions) measured with a spherical tip. Spatial deviation of *E* from the whole area median of *E* was for extra-nuclear cells regions smaller than 20%.

Small DMSO molecules enter even the nuclei of the cells and affect the freezing process of the cells so that the median of the stiffness is higher (compared to PEG treated frozen/thawed cells), cell nuclei is softest part of the cell immediately after thawing. The difference in stiffness between the core and the edge of DMSO treated/thawed cells was reduced within 2.5 h after thawing.

## Conclusions

Using AFM we studied stiffness of DMSO or PEG treated cryopreserved cells. Cryoprotectants used were of two types: penetrating both cell membrane and nuclear membrane (DMSO) or not penetrating even cell membrane (PEG 1500). The median stiffness of PEG treated frozen and thawed cells was lower compared to DMSO treated cells stiffness. PEG-cryopreserved cells viability was lower than DMSO treated cryopreserved cells. PEG treated cells were more round and taller immediately after thawing, with homogeneous spatial distribution of stiffness.

On the contrary, DMSO treated cells had considerably higher viability after thawing than PEG treated cells and cryopreserved stiffness maps of thawed DMSO treated cells were not homogeneous. Immediately after thawing DMSO treated cell nuclei are typically the softest part of the cells. The cytoskeleton is also clearly distinguishable in the modulus maps due to its comparatively higher stiffness. From 0.5 till 4.5 h after thawing, the spatial variation of stiffness of the DMSO-treated cells' surface gradually decreased. The median of the DMSO treated cryopreserved cells stiffness is higher than PEG treated cryopreserved cells. The selection of proper freezing medium has effect on cytoskeleton remodeling after freeze/thaw cycle, as DMSO seems to have protecting abilities toward actin filaments, while non-penetrating PEG protection of these molecules is insufficient and connected with longer remodeling time and higher elasticity of the cell.

We showed that AFM can be used as technique for investigation of cryopreserved cells surfaces state and development *ex vivo*. Our results offer a new perspective on the monitoring and characterization of frozen cells' recovery by measuring their elastic response to external mechanical stimuli. This may lead to a new and detailed way of investigating the post-thaw development of cryopreserved cells which allows to distinguish between different cell parts.

## Data availability statement

The datasets of the raw data (raw force map files recorded by the AFM microscope, force map files processed by AtomicJ software, fluorescence stack images and live cell imaging video recorded by confocal microscope) for this study can be found in the Zenodo file repository under following link: https://doi.org/10.5281/zenodo.1209827. Amount of the data files stored here exceeds 40 GB.

## Author contributions

MG AFM data processing and interpretation, writing parts of the manuscript. IK project initiation, writing parts of the manuscript. PS, MP, and VR writing parts of the manuscript. SJ cell culture and freezing/thawing manipulation, Life imaging, actin data interpretation, and manuscript writing. JP AFM force nanoindentation process, fluorescence stack imaging, data processing and interpretation, manuscript writing.

### Conflict of interest statement

The authors declare that the research was conducted in the absence of any commercial or financial relationships that could be construed as a potential conflict of interest.
