# Supplementary material for: AFM Monitoring the Influence of Selected Cryoprotectants on Regeneration of Cryopreserved Cells Mechanical Properties
Source: Front Physiol. 2018 Jun 29;9:804. doi: 10.3389/fphys.2018.00804 (PMC6034176; doi:10.3389/fphys.2018.00804)
Supplement: Supplementary file 3 [file Data_Sheet_1.docx]

Supplementary Material

AFM monitoring the influence of selected cryoprotectants on regeneration of cryopreserved cells mechanical properties

Martin Golan, Šárka Jelínková, Irena Kratochvílová, Petr Skládal, Martin Pešl, Vladimír Rotrekl and Jan Přibyl*

*** Correspondence:** Corresponding Author: pribyl@nanobio.cz

**List of figure captions**

[**Supplementary Figure 1:** Preview of live imaging videos attached as supplementary files to the manuscript. Post-thawing development of the cells cryo-protected with either DMSO (left) or polyethylene glycol (Mw 1500; PEG1500). 3](#_Toc515058293)

[**Supplementary Figure 2:** Effect of DMSO used as a cryoprotectant on the biomechanical and structural properties of living fibroblast cells. In the upper box, stiffness map (left), cell height map (images in the middle) and fluorescence images (right) of the cell at beginning and at the end of the time period of the measurement (4 hours). The lower box contains graphs that show median and mean value of elasticity over time (left), right: median value of elasticity in lower (0-50% of the full cell height), and higher (50-100% of the full cell height) part of cell over time. 4](#_Toc515058294)

[**Supplementary Figure 3:** AFM maps of Young’s Modulus (left), height (column of image in the middle) and fluorescence images of cytoskeleton (right) of polyethylene glycol (Mw 1500, PEG1500) treated frozen/thawed cells during monitoring of the post-thawing process. Each step shows a change of the cell biomechanical and structural properties during 60 minutes of the cell life. Polyethylene glycol (Mw 1500) treated cells both cytoskeleton and cell nuclei became softer and the height of the cell increased. 5](#_Toc515058295)

**List of table captions**

**Supplementary Table 1** Viability of cells prior to and after freezing/thawing with DMSO, PEG-1500 or no cryoprotectant, respectively. Viable cells were identified using flow cytometry (negativity for Annexin-V and 7-AAD).

**Supplementary Table 2**: Viability of cells at selected time points after freezing/thawing with DMSO or PEG-1500. Viable cells were identified using hematocytometer (negativity for TrypanBlue). The percentages are expressed relative to the size of the original cell population prior to freezing. Losses therefore include cells that died during freezing or didn’t sufficiently adhere to the dish within 30 minutes of culturing after thawing. For either treatment, the cell viability doesn’t significantly decrease during the AFM monitoring.


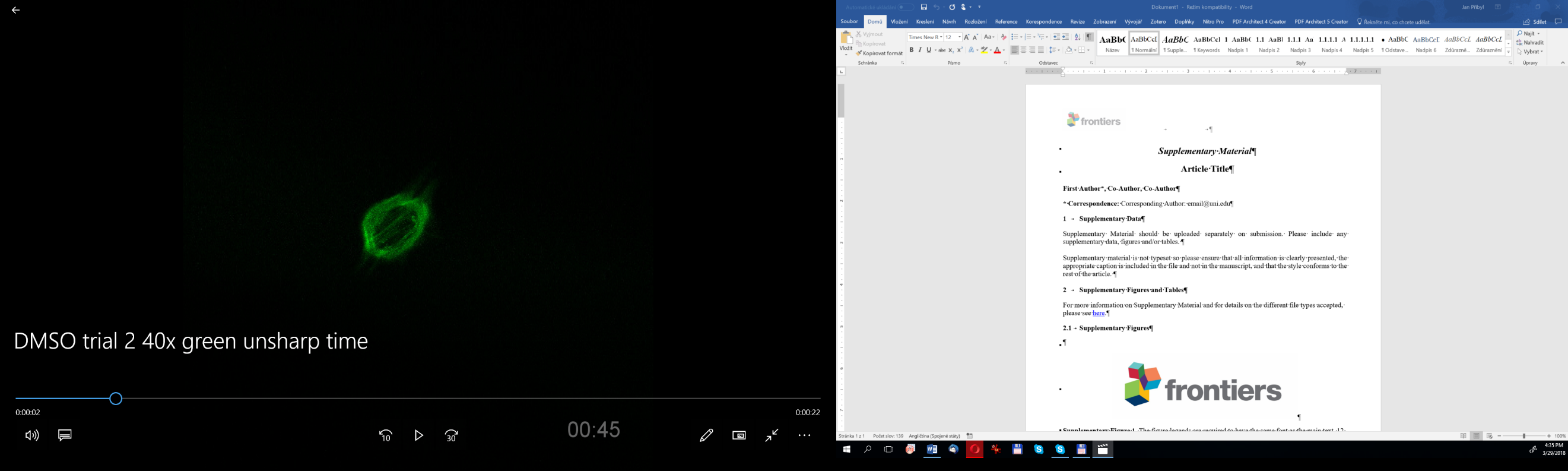

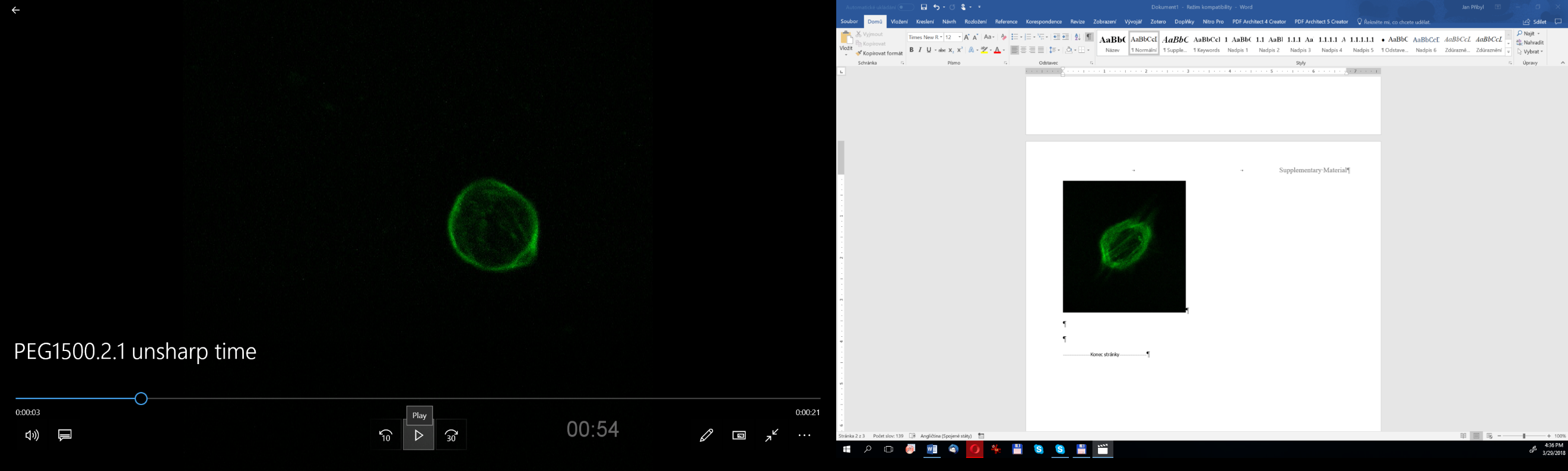


DMSO

PEG1500

**Supplementary Figure 1:** Preview of live imaging videos attached as supplementary files to the manuscript. Post-thawing development of the cells cryo-protected with either DMSO (left) or polyethylene glycol (Mw 1500; PEG1500).


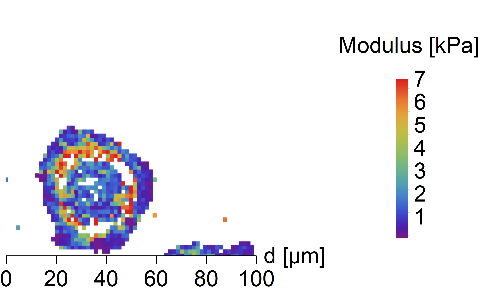

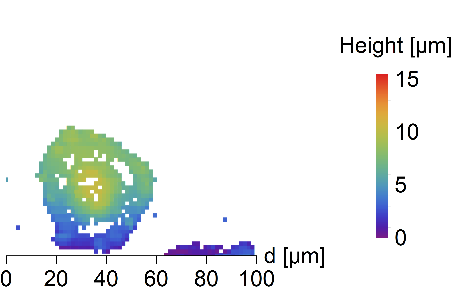


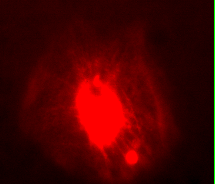


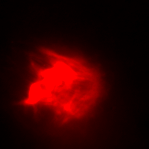

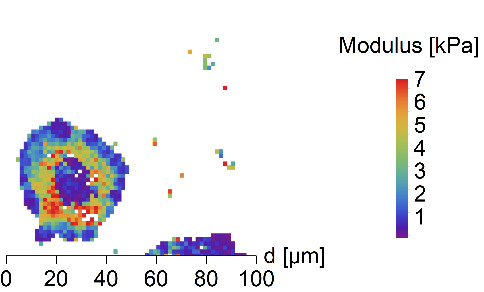

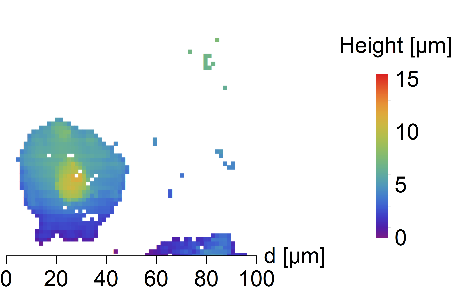


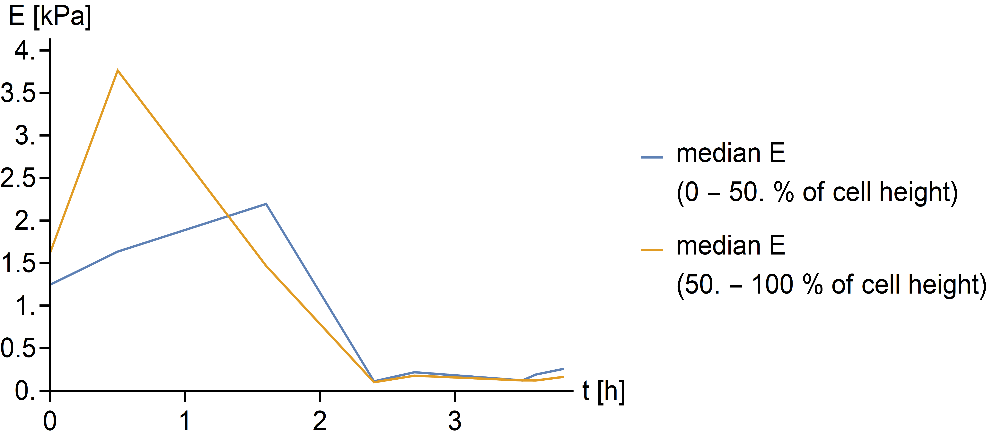

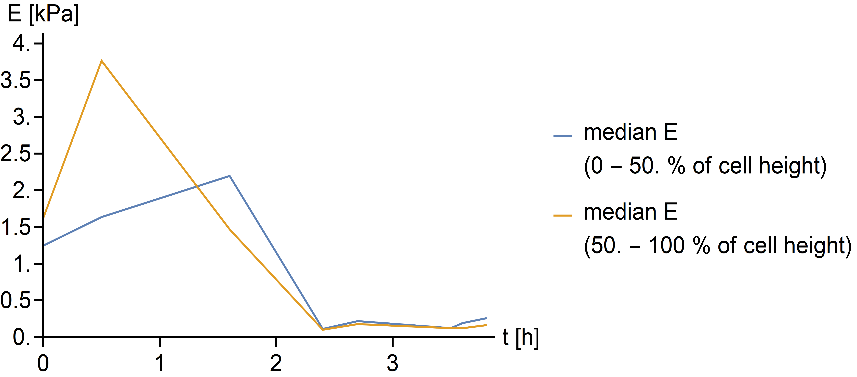

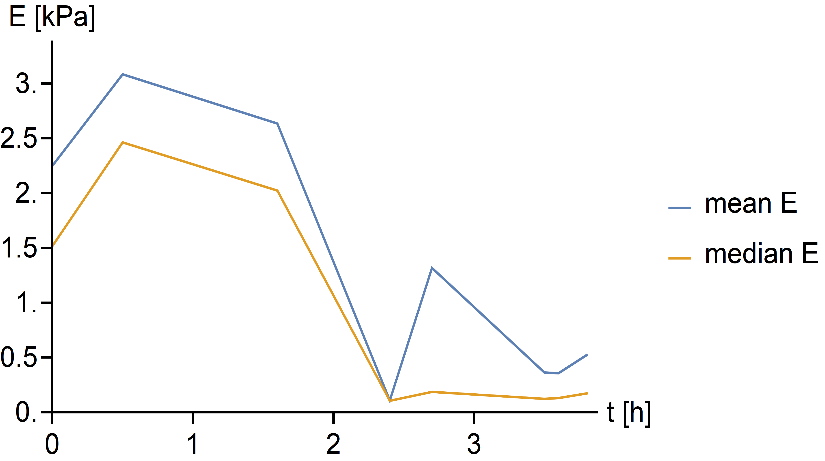

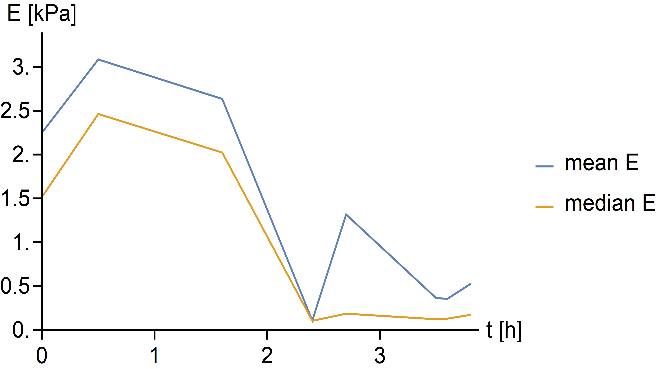


**Supplementary Figure 2:** Effect of DMSO used as a cryoprotectant on the biomechanical and structural properties of living fibroblast cells. In the upper box, stiffness map (left), cell height map (images in the middle) and fluorescence images (right) of the cell at beginning and at the end of the time period of the measurement (4 hours). The lower box contains graphs that show median and mean value of elasticity over time (left), right: median value of elasticity in lower (0-50% of the full cell height), and higher (50-100% of the full cell height) part of cell over time.


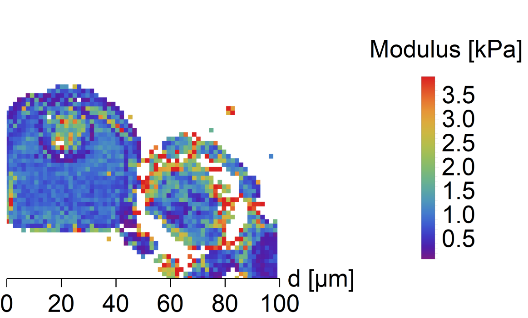

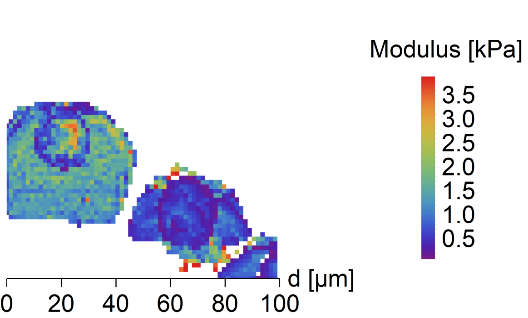

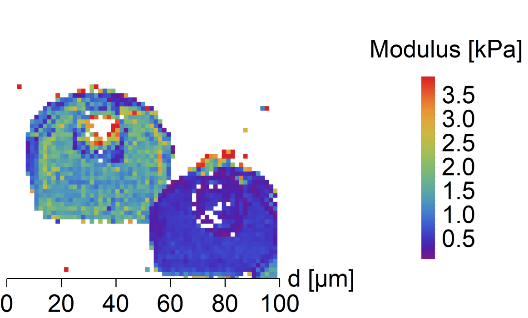

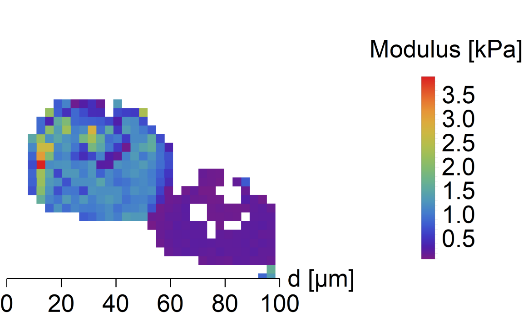

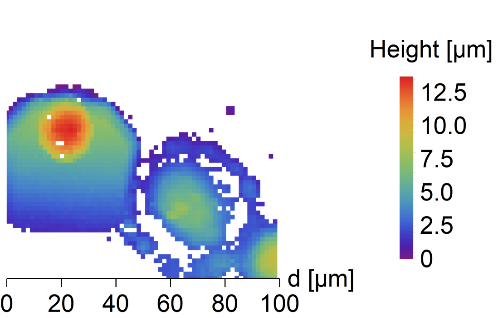

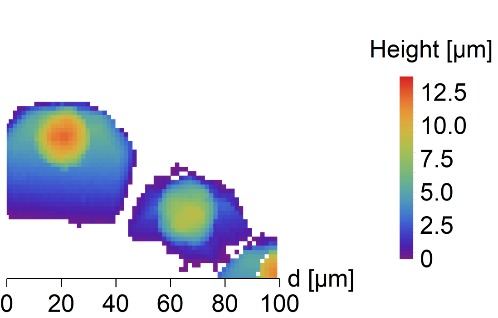

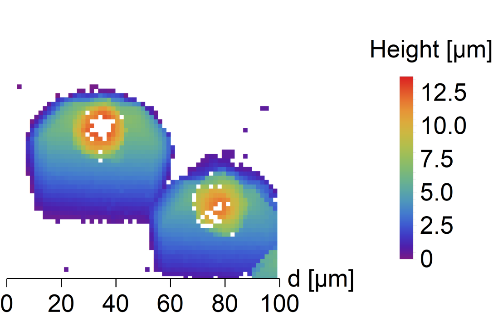

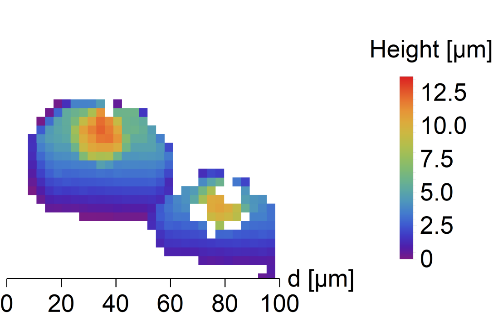

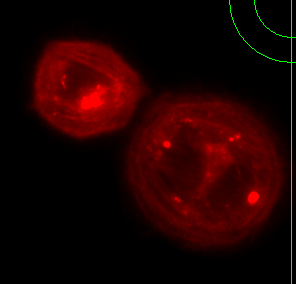

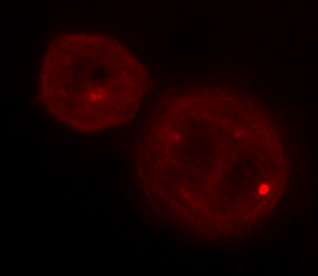

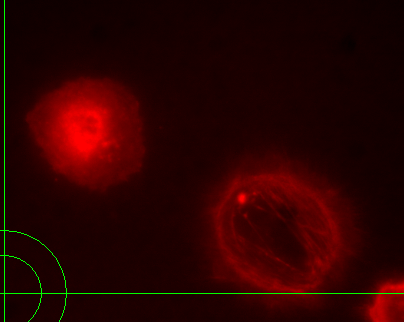

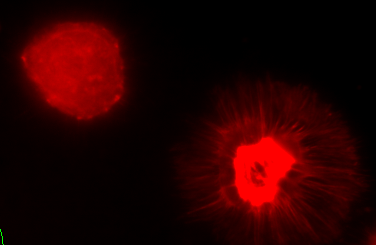


**Supplementary Figure 3:** AFM maps of Young’s Modulus (left), height (column of image in the middle) and fluorescence images of cytoskeleton (right) of polyethylene glycol (Mw 1500, PEG1500) treated frozen/thawed cells during monitoring of the post-thawing process. Each step shows a change of the cell biomechanical and structural properties during 60 minutes of the cell life. Polyethylene glycol (Mw 1500) treated cells both cytoskeleton and cell nuclei became softer and the height of the cell increased.

Supplementary Table 1 Viability of cells prior to and after freezing/thawing with DMSO, PEG-1500 or no cryoprotectant, respectively. Viable cells were identified using flow cytometry (negativity for Annexin-V and 7-AAD).

| Cell viability [%] (Annexin-V/7-AAD) | | Non-frozen cells | Frozen/thawed cells |
| --- | --- | --- | --- |
| Treatment | No cryoprotectant | 96 | < 5 |
|  | DMSO | 93 | 87 |
|  | PEG-1500 | 92 | 56 |

**Supplementary Table 2**: Viability of cells at selected time points after freezing/thawing with DMSO or PEG-1500. Viable cells were identified using hematocytometer (negativity for TrypanBlue). The percentages are expressed relative to the size of the original cell population prior to freezing. Losses therefore include cells that died during freezing or didn’t sufficiently adhere to the dish within 30 minutes of culturing after thawing. For either treatment, the cell viability doesn’t significantly decrease during the AFM monitoring.

| Cell viability [%] (Trypan blue) | Time after freezing/thawing | | | | |
| --- | --- | --- | --- | --- | --- |
|  | 0.5 h | 1.5 h | 2.5 h | 3.5 h | 4.5 h |
| DMSO | 61 | 61 | 60 | 58 | 60 |
| PEG-1500 | 32 | 31 | 30 | 31 | 30 |
