# Supplementary figures and images for: AFM Monitoring the Influence of Selected Cryoprotectants on Regeneration of Cryopreserved Cells Mechanical Properties
Source: Front Physiol. 2018 Jun 29;9:804. doi: 10.3389/fphys.2018.00804 (PMC6034176; doi:10.3389/fphys.2018.00804)

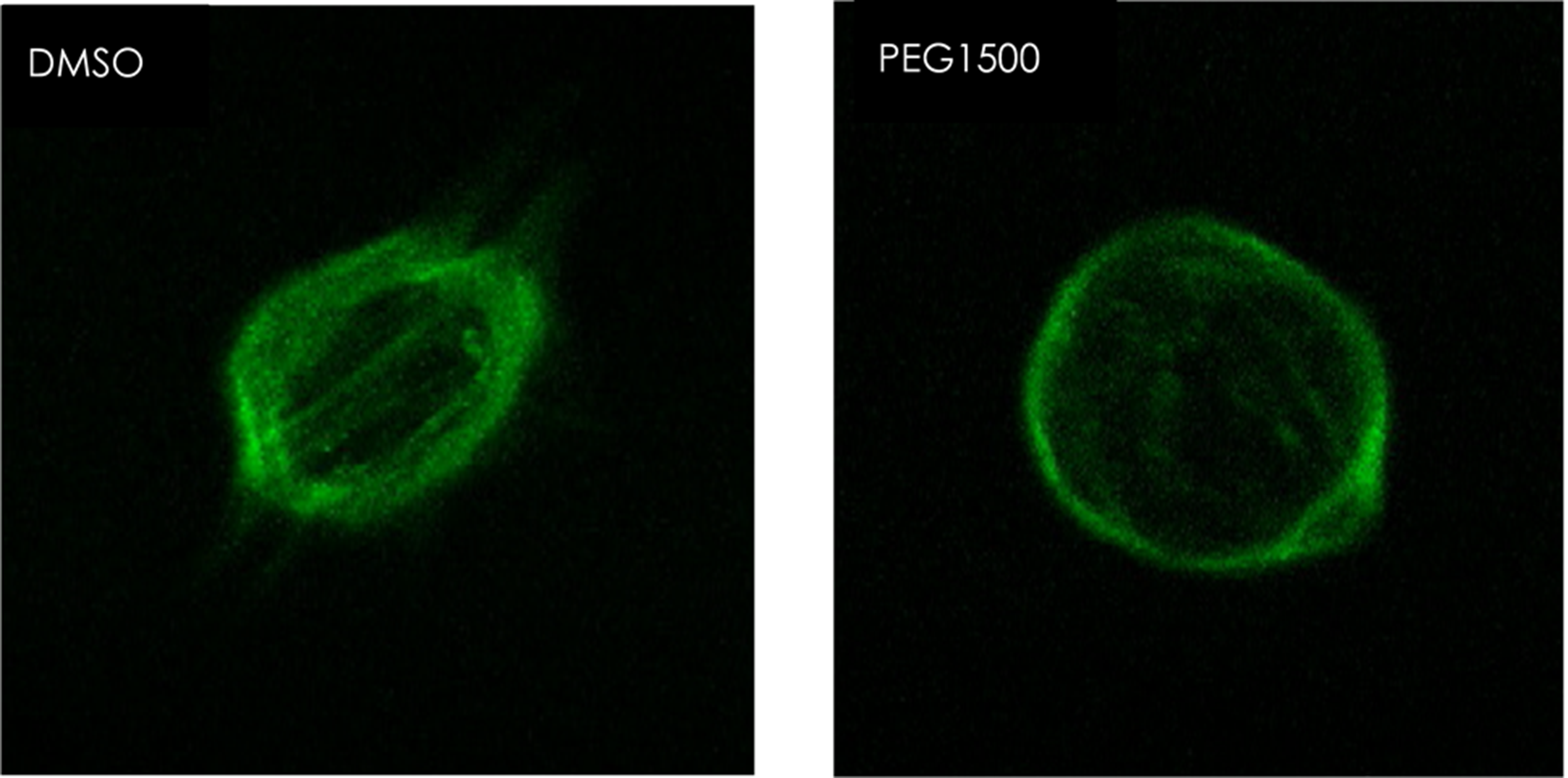

Supplement: Supplementary file 4 [file Image_1.tif]
